# Intelligence

**Published:** 1830-04

**Authors:** 


### INTELLIGENCE.

### ROYAL COLLEGE OF SURGEONS.

*Lectures on Anatomy and Surgery, by* Professor Guthrie.

The high reputation for practical knowledge which Mr. Guthrie possesses, combined with his eloquent and animated style of imparting his information to others, renders these lectures unusually interesting. The subjects which he has already considered in the anatomical part of his course, were, first, on the Bones of the Pelvis, and on the difference between the pelves of the various races of man, in which a comparison was drawn between them and the lower animals, and the gradation from the European downwards well illustrated. On the difference between the pelvis of the virgin and the matron, Mr. Guthrie was rather amusing, showing that, after the lapse of a couple of thousand years, the chastity of a lady might, by this rule, be doubted, that had heretofore never been suspected. The second lecture was on the Kidney, its minute structure and secretion. The third, on the Ureters and Bladder. The fourth, on the Prostate Gland, and on the side view of the Pelvis, male and female. The fifth, on the Urethra and internal parts: the whole anatomy being rendered subservient to observations on the use and proper methods of introducing instruments into the bladder. Among many points of great interest, Mr. Guthrie mentioned the occurrence of a stone sticking in the orifice of one of the ureters, (and showed the preparation,) which had been constantly felt, and the patient was to have been operated upon, if death had not prevented it. This peculiarity, combined with a tap or blow often felt on the catheter when the urine was drawn off, and which occurred from the sudden contraction of the lower and posterior part of the bladder, caused, he suspected, in some cases operations to be performed, when no stone could be discovered. The stone would be in the same state as if sacculated, and it led to the practical inference that the stone should always be felt above the wound and not below it, and that the surgeon should not be content to feel it, but he should also be certain that he could move it.

---

### NEW REGULATIONS OF THE SOCIETY OF APOTHECARIES.

*Extract from Resolutions of the Court of Assistants of the Society of Apothecaries.*

Resolved: That the Society's garden at Chelsea be open every Wednesday during the months of May, June, July, August, and September, from nine o'clock in the morning until twelve at noon, and that admission be given to all such medical students as are pupils to the established professors and lecturers in the metropolis, whether upon medicine, chemistry, materia medica, or botany, and also to the apprentices of the several members of the Society.

That there be every week a demonstration of the plants contained in that department of the garden appropriated to plants belonging to the materia medica, and of such other plants as the demonstrator may think proper: such demonstration to commence at ten o'clock punctually; and that, after such demonstration is finished, there be a lecture delivered by the demonstrator in some part of the building attached to the garden, upon one or more of the following subjects, so as to form, during each summer season, a regular course of botanic study; viz.

1. The different systems of botany, both natural and artificial, particularly those of Linnæus and Jussieu.

2. The structure and growth of plants.

3. The different parts of plants, with their descriptions and uses in the process of vegetation.

4. The natural and chemical analysis of vegetable matter.

5. The medical uses of the most important articles in the materia medica, with observations on the best modes of preparing them. These remarks may be made either at the lectures or at the demonstrations, at the discretion of the lecturer.

That the conducting these demonstrations and lectures be committed to the Society's demonstrator of botany, and that the monthly lectures hitherto delivered by him at the gardens be discontinued, as merging and more effectually provided for in the lectures now proposed to be adopted.

That in order to give encouragement to diligence and talent, there be an annual examination of such students as may think proper to become candidates for the prizes intended to be given on these occasions. The examinations to be upon some or all of the subjects stated in the foregoing series of lectures, as well as upon their skill in the nomenclature of plants. No person to be admitted a candidate who has not attended these lectures and demonstrations at least eighteen days in one summer, or thirty days in two succeeding summers; nor shall any prize be awarded unless this examination be performed to the complete satisfaction of the examiner or examiners for the time being.

To prevent partiality or undue preference, no public professor or lecturer, whose pupils are admitted to the garden, can be appointed an examiner.

The apprentices to members of this Society having an annual opportunity of being candidates for prizes upon the ancient establishment, cannot be admitted candidates on these occasions, either during the period of their apprenticeship, or subsequently to the conclusion of it.

That two medals, the one being of gold, of ten guineas value, and the other of silver or bronze, be annually awarded to the two candidates who shall have passed the best and second best examination in manner hereinbefore mentioned ; but no medal is to be given unless, in the opinion of the examiner or examiners, the candidates shall be deemed deserving of it.

The beadle, or some proper person, is to attend at the garden on each day of admission, to receive the visitors, and to enter or cause their names, and the names of their tutors, to be entered regularly, in a book to be provided for that purpose; and also to note therein any misconduct or breach of established regulations which may come to his knowledge during such attendance, giving information thereof to the Master and Wardens.

That the following be the regulations for the admission of students:

"It is intended that admission shall be given to all such medical students as are pupils to the established professors and lecturers in the metropolis, whether upon medicine, chemistry, materia medica, or botany; such students to apply at least three days prior, at the beadle's office, in Apothecaries' Hall, for tickets of admission for that purpose, which the Master and Wardens will grant to such persons as they may think proper.

"In order that the Master and Wardens may be enabled to exercise suitable discretion in granting such tickets, each student must leave with the beadle a letter of recommendation from his tutor, stating that such student has been attentive to his studies, and is, in his opinion, desirous of improving himself in the science of medical botany.

"That a ticket be given to each student, and that such ticket be renewed annually."

(By order,) EDMUND BACOT, Clerk.

*Apothecaries' Hall; Feb.* 1, 1830.

### BOTANICAL LECTURES.

Mr. Gilbert Burnett will commence his Botanical Lectures, at the Royal Institution, on the last Wednesday in April; subjects for this season: Illustrations of the Philosophy of System; of the Principles of Arrangement in Natural History in general; and of Methodical Distribution, as practically applied to the Vegetable Kingdom in particular: and in the Theatre of Anatomy, Great Windmill street, on the second Tuesday in May; which latter course will include a complete series of instruction in Physiological and Systematic Botany, with Herbarizings and Demonstrations, to ensure a knowledge of the medicinal and indigenous plants.

### HUNTERIAN MEDAL.

A gold medal, of the value of ten guineas, having been placed at the disposal of the Council of the Hunterian Society, for the best Essay on any subject selected by them, they have chosen for the current year "The Nature and History of Tubercular Formations." Essays must be delivered before the 1st of December: they must be addressed to the Secretaries; and the name of the author, with a motto corresponding with one prefixed to the essay, must be contained in a sealed packet.

The merits of the essays will be adjudged by the Council, and the prize presented at the anniversary meeting in February.

J. T. CONQUEST, } *Secretaries.*

WM. COOKE, }

18, *Aldermanbury; March* 10, 1830.

### LITERARY NOTICE.

In the ensuing spring will be published the first Number of the "North of England Medical and Surgical Journal, and Topographical and Statistical Record," which will be continued quarterly: four Numbers will form a handsome octavo volume.

### M. CHABERT, AND HIS ANTIDOTE FOR PRUSSIC ACID, &c.

Notwithstanding the repulses with which this claimant for scientific fame has recently had to contend, he still remains in the field, and bold in protestation. We did not imagine that we should again have been called upon to refer to his experiments, but as, since our last Number, he has done some deeds which have excited the attention of many, and perhaps produced conviction in a few, we are bound, as honest recorders, to state the facts of the case.

In compliance with an invitation from M. Chabert, we attended a meeting in St. James's street, about a fortnight ago. There were present a few whom we did know, and many whom nobody knew: the latter appeared, from their premature plaudits, to be very zealous in the cause. The amusements of the day commenced by M. Chabert's swallowing fifty grains of phosphorus. In the performance of this feat there was neither trick nor stratagem; the dose was fairly given, and fairly swallowed: but it is to be stated that the phosphorus was in lumps of several grains, and that, taken in this form, many hours would elapse before it would begin to produce any injurious effect. Orfila informs us that phosphorus given *in substance* to animals destroys by causing an *indolent* inflammation of the alimentary canal. A powerful emetic would, therefore, be the only antidote required in this case. It is true that M. Chabert offered to dine with any gentleman present, and remain with him the whole evening, to prove that he would not have recourse to an emetic for the purpose of getting rid of the material he had swallowed. The challenge for an invitation to dinner was not, however, accepted We may remark, that phosphorus in substance has often been taken in large doses. VATER took ten grains; DESBOIS DE ROCHEFORT,* a writer on Materia Medica, whose judgment ranks high on the continent, fixes the dose, *as a medicine*, from four to ten grains.† If M. Chabert will take twenty, or even ten, grains of phosphorus dissolved in oil or ether, we shall then have more confidence in the preservative powers of his antidote.

The next scene consisted in administering prussic acid to dogs; and after much delay, and various attempts to satisfy the company by very unsatisfactory and delusive experiments, the following experiment was proposed, and very unwillingly acceded to by M. Chabert, who confessed that he had no confidence in his antidote unless it were given *at the very moment the poison was placed upon the tongue of the animal;* when, in fact, the constitution would not be affected by its deleterious properties, and when, consequently, no other antidote could be required than a plentiful supply of liquid to wash it out of the mouth of the creature. This, indeed, was evidently the only object; for we particularly remarked, in his previous experiments, that M. Chabert dashed his "antidote" carelessly upon the tongue, and that he appeared quite indifferent whether any of it were swallowed or not. But to proceed: Two dogs were selected, apparently of equal strength; to one, about twelve minims of diluted prussic acid were given, which were nearly equivalent to two drops of the concentrated. The animal died in seven minutes, after having suffered from violent convulsions. The same quantity was given to the other dog; and at the expiration of thirty seconds, when the lower limbs were rigidly extended, a concerted signal was given, upon which M. Chabert administered the antidote, and frequently applied a liquid to the nostrils, which in appearance and smell resembled the aromatic spirit of ammonia. For some time the life of the animal seemed to be in much jeopardy: violent convulsions and great exhaustion ensued; but after much suffering he recovered, as much to the astonishment of M. Chabert as any body else, for he had previously declared that he did not believe the experiment would succeed, but that he would "trust to Providence, and take his chance."

Now, to us this solitary fact is far from satisfactory: in order to produce conviction in our minds, the same experiment must be repeated upon several dogs; and further, those which are to die, and those which are not to die, must be treated exactly in the same manner, with the exception of giving the antidote. We doubt very much whether the dog which was destroyed in the first experiment would not have survived, if he had been as liberally supplied with ammonia and fresh air as the second.

A few days after the meeting, a paper was sent to us for signature, in which the above experiments were related. We declined the honour of appearing in the list of M. Chabert's supporters: we remain among the *non-contents.*

The public will shortly be edified by M. Chabert's biography. He states

* Dict. des Sc. Med. t. xli. p. 518.

† Dr. CHRISTISON remarks, that the uncertainty and occasional severity of the operation of phosphorus have very properly expelled it from modern pharmacopœias. (*Treatise on Poisons*, p. 135.)

that he came into possession of his invaluable antidote in the following manner: In his infancy, while under the care of a priest, his curiosity was excited by observing that his tutor was much occupied in some very deep and secret study in his chamber. To these cogitations nobody was admitted; but young Chabert, like Paul Pry, put his eye to the keyhole, and ascertained that one particular bottle was frequently examined, and carefully concealed. He *borrowed* this mysterious bottle, and carried it to a learned chemist, who analysed the contents, and detected its composition and virtues. Why the chemist did not make use of so important a discovery for his own advantage, we know not. Chabert, still very young in age, but old in science, forthwith started on a journey to exhibit the virtues of his antidote. His success, he assures us, has been quite marvellous in every part of the globe except England.

### METEOROLOGICAL JOURNAL,

By Messrs. HARRIS and Co. Mathematical Instrument Makers, 50, High Holborn.

| February | Rain gauge. | Moon. | Thermom. | | | Barometer. | | De Luc's Hygrom. | | Winds. | | Atmospheric Variations. | | |
|---|---|---|---|---|---|---|---|---|---|---|---|---|---|---|
| | | | 9 a.m. | max. | min. | 9 A.M. | 10 P.M. | 9 A.M. | 10 P.M. | 9 A.M. | 10 P.M. | 9 a.m. | 2 p.m. | 10 p.m. |
| 20 | | | 32 | 37 | 32 | 29.72 | 29.68 | 64 | 61 | WNW | W | Foggy | Fine | Fine |
| 21 | | | 35 | 39 | 30 | .63 | .58 | 63 | 65 | W | W | Fine | Rain | Cloudy |
| 22 | | | 35 | 48 | 34 | .61 | .53 | 65 | 68 | WNW | SW | Foggy | Fine | Rain |
| 23 | .05 | ● | 48 | 53 | 47 | .57 | .76 | 70 | 70 | WSW | WSW | Rain | Cloudy | Show'ry |
| 24 | | | 51 | 55 | 48 | .82 | .97 | 70 | 69 | WSW | WSW | Foggy | Fine | Fine |
| 25 | | | 53 | 55 | 50 | 30.00 | 30.00 | 69 | 68 | WSW | SW | Fine | — | — |
| 26 | | | 54 | 55 | 49 | 29.96 | 29.91 | 68 | 66 | WSW | SW | — | Cloudy | Rain |
| 27 | .03 | | 50 | 52 | 46 | .92 | .94 | 65 | 65 | W | WSW | — | Fine | Cloudy |
| 28 | | | 51 | 54 | 45 | 30.05 | 30.17 | 65 | 65 | W | W | — | Cloudy | Fine |
| Mar. | | | | | | | | | | | | | | |
| 1 | | ☽ | 50 | 55 | 49 | .21 | .24 | 65 | 65 | W | W | Foggy | Fine | — |
| 2 | | | 52 | 55 | 48 | .23 | .15 | 63 | 63 | WSW | W | Fine | — | — |
| 3 | | | 50 | 53 | 35 | .08 | 29.91 | 60 | 58 | S | S | — | — | — |
| 4 | | | 41 | 50 | 36 | 29.85 | .85 | 58 | 60 | S | ESE | — | — | — |
| 5 | | | 39 | 43 | 35 | .90 | .87 | 60 | 62 | SE | ESE | — | — | — |
| 6 | | | 40 | 50 | 32 | .87 | .91 | 62 | 62 | ESE | ESE | — | — | — |
| 7 | | | 36 | 43 | 35 | .91 | .78 | 63 | 63 | E | E | — | — | — |
| 8 | | ○ | 41 | 45 | 40 | .56 | .42 | 64 | 60 | E | SE | Foggy | Fine | Cloudy |
| 9 | | | 44 | 48 | 44 | .54 | .58 | 60 | 64 | S | S | Cloudy | Sleet | Cloudy |
| 10 | | | 47 | 55 | 45 | .53 | .82 | 67 | 66 | S | WSW | Cloudy | Fine | Fine |
| 11 | | | 53 | 57 | 45 | .98 | .88 | 66 | 65 | W | WNW | Fine | — | — |
| 12 | | | 52 | 59 | 38 | 30.03 | 30.12 | 63 | 61 | WNW | W | — | — | Sleet |
| 13 | | | 50 | 57 | 36 | 29.81 | 29.47 | 55 | 55 | WNW | WSW | — | — | Fine |
| 14 | | | 44 | 52 | 39 | .34 | .34 | 57 | 57 | SW | SW | — | — | — |
| 15 | | | 45 | 49 | 37 | .50 | .69 | 58 | 58 | SW | W | — | Rain | — |
| 16 | .08 | | 44 | 55 | 42 | .67 | .89 | 58 | 60 | WSW | W | — | Hail | — |
| 17 | | ☾ | 55 | 56 | 47 | 30.01 | 30.10 | 60 | 60 | W | W | Cloudy | Fine | — |
| 18 | | | 54 | 59 | 44 | .12 | .04 | 60 | 57 | W | WSW | Fine | — | — |
| 19 | | | 52 | 60 | 43 | .00 | .07 | 57 | 56 | WSW | WSW | — | — | — |

The quantity of Rain cannot be given for February, on account of the frost.

